# Impact of a police safeguarding program on reducing dementia-related missing incidents in the United Kingdom

**DOI:** 10.1093/geroni/igaf132

**Published:** 2026-03-04

**Authors:** Sol Morrissey, Stuart King, Ben Au-Yeung, Michael Hornberger

**Affiliations:** Department of Biomedical Engineering, University of Calgary, Calgary, Alberta, Canada; Norwich Medical School, University of East Anglia, Norwich, United Kingdom; Avon and Somerset Police, Police and Fire Headquarters, Portishead, North Somerset, United Kingdom; Norwich Medical School, University of East Anglia, Norwich, United Kingdom; Norwich Medical School, University of East Anglia, Norwich, United Kingdom; Department of Clinical Neurosciences, University of Southampton, Southampton, United Kingdom

**Keywords:** Cognitive impairment, Missing person, Emergency services, Intervention, Public health

## Abstract

**Background and Objectives:**

People living with dementia are at increased risk of missing episodes, which can have serious safety consequences for the individual as well as increasing burden for families, emergency services, and care services. A UK police safeguarding scheme was developed in response to reduce the risk of missing incidents through proactive risk management and early intervention. This study evaluates whether the safeguarding scheme effectively reduces the risk of missing incidents for individuals taking part in the scheme.

**Research Design and Methods:**

We conducted a retrospective cohort study using a police database of 846 individuals living with dementia taking part in the safeguarding scheme. Descriptive statistics and proportion comparisons were used to evaluate changes in missing incident characteristics before and after joining the scheme, stratified by risk level and dementia subtype.

**Results:**

We found that there were fewer missing incidents and fewer individuals with a recorded missing episode after joining the safeguarding scheme. Individuals with first missing incidents occurring after joining the scheme were found significantly faster (2.73 hours) than those with a first incident occurring before joining the scheme (5.39 hours). Among those identified as high-risk—individuals with a previous missing incident—81.21% did not go missing again after participating in the scheme. Individuals with Alzheimer’s disease were more likely to go missing after taking part in the safeguarding scheme than those with vascular dementia. While individuals with a history of missing incidents remained at higher risk, the majority did not go missing again after joining the scheme.

**Discussion and Implications:**

Overall, the safeguarding scheme was effective in reducing the rate of missing incidents among people with dementia. These findings promote the proactive use of police safeguarding programs and suggest that widespread implementation could improve safety and independence for people living with dementia.

Innovation and Translational Significance: People with dementia are at high risk of missing incidents, presenting significant safety concerns. This study evaluates the impact of a UK-based Police Dementia Safeguarding Scheme, which aims to reduce dementia-related missing incidents. We establish that the scheme is effective in reducing the number of dementia-related missing incidents and the duration of missing incidents, with 81% of high-risk individuals not reporting a missing episode after joining. Broader implementation of the Dementia Safeguarding Scheme may therefore enhance the safety of people living with dementia, reduce carer and emergency service burden, and support independent living and aging in place.

Missing incidents are a critical concern for individuals with dementia, their families, and caregivers, with approximately 40,000 people with dementia reported missing for the first time each year in the United Kingdom (Alzheimer’s Association, 2022; NPIA, 2011). These incidents can have devastating consequences, including harm or death due to exposure and dehydration (Larsson et al., 2025; Perez et al., 2024; Rowe et al., 2010; White & Montgomery, 2015). Beyond the physical risks, missing episodes significantly distress carers and family members, often leading to a myriad of challenges. Repeated missing incidents have been associated with a seven-fold increase in care home admissions for individuals with dementia, as families face mounting difficulties in ensuring their safety (Yatawara et al., 2017). Consequently, these incidents often restrict the independence of individuals with dementia, as they may no longer be permitted to venture out alone or are placed in institutional care settings. Promoting the independence of people with dementia while safeguarding their well-being is a critical priority for individuals, families, and policymakers—see also the UK Chief Medical Officer’s annual report (Whitty, 2023).

Most individuals living with dementia go missing during brief periods of unsupervised activity while engaged in typical daily routines. These incidents are challenging to predict, occurring without clear warning signs, and can result from contextual, situational, environmental, and cognitive factors (Ferguson, 2022; Perez et al., 2024; Puthusseryppady et al., 2019; Rowe et al., 2015). Despite potentially serious consequences of missing incidents, most missing incidents are underreported (Greene et al., 2019; Neubauer et al., 2021). Studies estimating the prevalence of missing incidents using secondary databases have also been constrained by small sample sizes, limiting the scope for generalizability and statistical analysis (Miguel-Cruz et al., 2024). Furthermore, a recent narrative synthesis found that there was limited evidence for recommending interventions for preventing people living with dementia from missing incidents (Emrich-Mills et al., 2021).

In most countries, police services are responsible for responding to missing persons. However, research on police involvement in dementia-related missing incidents is currently very limited (Neubauer et al., 2019), despite them being first responders to such events. Police services can vary in their approach to handling dementia-related missing incidents, with some services adopting proactive risk reduction strategies and others taking a more passive approach (Kikuchi et al., 2025).

This study addresses these shortcomings by evaluating a real-world safeguarding program currently employed by the Avon and Somerset Police in the United Kingdom, adopted by many police services across the United Kingdom. This study aims to conduct an impact assessment of the Avon and Somerset Dementia Safeguarding Scheme on reducing missing incidents and improving the safeguarding of individuals with dementia. The specific aims of the study are to establish (a) characteristics of individuals who take part in the safeguarding scheme; (b) whether taking part in the safeguarding scheme reduces the risk of missing incidents for individuals with a history of missing incidents; (c) whether taking part in the safeguarding scheme is associated with reduced duration of missing incidents; (d) whether the safeguarding scheme impacts locations where individuals are reported missing and found; and (e) whether the safeguarding scheme differs in effectiveness for different dementia diagnoses.

We hypothesize that (a) more individuals with Alzheimer’s disease (AD) will take part in the safeguarding scheme and show greater risk for missing incidents; (b) participants will experience fewer missing incidents and there will be fewer missing individuals after joining the safeguarding scheme; (c) the duration of missing incidents will be shorter for missing incidents taking place after joining the safeguarding scheme than before; (d) there will be no significant differences in the locations missing or locations found before or after taking part in the safeguarding scheme; and (e) the safeguarding scheme will be effective for all individuals living with dementia, but the most significant safeguarding improvement will be found in individuals with AD.

## Method

### Study design

This study employed a retrospective, observational design using secondary data from the Avon and Somerset Police records detailing their Dementia Safeguarding Scheme participant database. A total of 846 participants took part in the safeguarding scheme, with 255 overall missing incidents reported. Ethical approval for the study was provided by the Faculty of Medicine and Health Sciences Research Ethics Committee at the University of East Anglia (22/PR/0928).

### Avon and Somerset Police dementia safeguarding scheme

In 2015, assessments conducted by the Avon and Somerset Police highlighted a high volume of dementia-related missing incidents. In response, the scheme introduced a multifaceted safeguarding approach designed to reduce the likelihood of individuals with dementia going missing, while also alleviating pressures on police resources. The scheme involves three main components: (1) an online Herbert Protocol; (2) radio-frequency identification (RFID) assistance devices; and (3) community engagement initiatives. The Herbert Protocol is an online form used to record key information about individuals with dementia—such as physical description, medical needs, routines, and known locations—before they go missing. Linkage with Police systems enables immediate access during missing incidents, where information about the individuals can help efficiently guide missing person searches. Individuals completing an online Herbert Protocol registration were asked whether they wished to include an order request for an assistive RFID device to support locating the individual if they become lost. At the time of the study, applicants could request near-field communication (NFC) devices via the order form, while GPS devices were allocated internally by police due to limited availability (maximum of 30 devices). Following the study period (from December 2024 onwards), Life360 Bluetooth Tracking Tiles were made available free of charge through the registration process (see Supplementary Table 1 for detailed device information). Finally, community engagement initiatives, led by the Avon and Somerset Dementia Forum, raise awareness through updated news and initiatives and promote the development of dementia-friendly communities.

The scheme incorporates an alert flagging system integrated into police command, control, and record management systems. This system automatically flags reports involving individuals registered with the safeguarding scheme, ensuring officers are immediately aware when a case involves a vulnerable person with dementia. Such alerts enhance decision-making in managing Threat, Harm, and Risk, and enable police to prioritize attendance, apply tailored safeguarding measures, and respond more effectively at the point of contact.

### Safeguarding scheme database

Access to the database was provided by Inspector Stuart King KPM from Avon and Somerset Police in June 2023. Information within the database was made de-identifiable prior to analysis, so as not to allow identification of participating individuals. Variables within the database included date of birth, dementia diagnosis, date joining the safeguarding scheme, date and time of missing incident(s), date and time of being found, locations missing (home, public, care home, hospital), and locations found (home, public, care home, hospital), and final data census date. Participants were initially categorized into high-risk (previous history of ≥1 going missing incidents before joining the safeguarding scheme) and low-risk (no previous history of going missing before joining the safeguarding scheme) groups. The high-risk group informs as to whether the Dementia Safeguarding Scheme reduces missing incident recurrence, while the low-risk group informs as to whether the Dementia Safeguarding Scheme prevents incident onset.

### Statistical analysis

We initially removed data from 13 participants who had duplicate values, had no record of a niche tag provided, or were listed as having absconded. Three participants listed as having Korsakoff syndrome, lymphoma, and memory loss, as well as nine participants listed as having “undiagnosed dementia,” were excluded from analyses.

For analyses, we first characterized the time and seasonality of missing incidents. A chi-square goodness-of-fit test was used to assess whether missing incidents were more likely to occur in Spring (March, April, and May), Summer (June, July, and August), Autumn (September, October, and November), or Winter (December, January, and February). A chi-square goodness-of-fit test also assessed whether missing incidents were more likely to take place in the morning (05:01–13:00), afternoon/evening (13:01–21:00), or night (21:01–05:00). Bonferroni-adjusted post-hoc pairwise analysis of proportions compared differences between pairs of time categories. We then assessed the effectiveness of the safeguarding scheme for reducing missing incidents. Missing incidents were categorized by safeguarding status (before joining, after joining). A *t*-test was used to compare the number of missing incidents by safeguarding status. A chi-square test of independence was used to assess the likelihood of missing incidents after joining the safeguarding scheme between high- and low-risk groups. We then assessed whether there were differences in the duration of missing incidents within participants by safeguarding status. Only missing incidents with recorded date–time values for time missing and time found were included for analysis (233 missing incidents with calculable hours value out of 255 missing incidents overall). A *t*-test was used to assess the duration of missing incidents by safeguarding status. A *t*-test was also used to assess the difference in missing incident duration for high-risk individuals who recorded a missing incident after joining the safeguarding scheme. For further post-hoc analysis, we examined the difference in duration of only the first recorded missing incident between safeguarding status groups. To establish whether there were differences in the locations missing and locations found by pre- and post-safeguarding missing incidents, incident locations were categorized into four groups (home, public, care home, and hospital). For the analysis on location missing and found, the analysis only focused on data that had a known location (237 known incidents in total). Unknown location incidents were removed from analysis. A Fisher’s exact test was used to assess differences in locations missing or locations found between by safeguarding status due to small sample frequencies within public, care home, and hospital locations. We then assessed how missing incident characteristics differed across dementia diagnoses. For this analysis, only individuals with AD and vascular dementia (VD) were included as other dementia subtypes (frontotemporal dementia, Parkinson’s disease dementia, and dementia with Lewy bodies) did not have substantive sample sizes. Additionally, individuals with mixed dementia or unspecified dementia were removed from analyses due to the absence of a clear clinical diagnosis. *T*-tests were used to assess differences in the number of reported missing incidents between the AD and VD groups by safeguarding status, as well as the duration of missing incidents. A Pearson’s chi-squared test was used to assess whether there was a difference in the number of individuals who reported multiple missing incidents between the AD and VD groups.

For post-hoc analysis, we assessed how the duration of time participating in the program influenced missing incidents. To address potential time bias in the amount of time individuals had spent in the safeguarding scheme, we repeated our analyses on the number of missing incidents and the number of individuals with missing incidents, considering only individuals who had joined the safeguarding scheme at least 1 year prior to the data census collection date (June 8, 2023). This approach ensured that observed effects were not confounded by shorter exposure durations among individuals who had more recently enrolled in the safeguarding scheme. Previous research also demonstrates that repeated missing incidents were found to take place typically after an 11-month period (Miguel-Cruz et al., 2024), supporting the rationale for selecting a 1-year cutoff to capture the full potential impact of the intervention.

## Results

### Characteristics of safeguarding scheme participants

The average age of safeguarding scheme participants when joining was 80.00 (*SD* = 9.96). The majority of individuals within the scheme had no recorded history of going missing (78.49%), with 182 individuals having a previous police-recorded missing incident (21.51%). Of the individuals who had a history of going missing, 149 recorded a missing incident before joining the scheme (81.87%), and 61 recorded a missing incident after joining the scheme (33.52%). A total of 149 participants were therefore classified as high risk when joining the safeguarding scheme (see Table 1).

**Table 1.** Demographic characteristics of individuals participating in the safeguarding program (*N *= 846).

Variable	Mean (*SD*)	%	*N*
**Age of participants when joining program**	80.00 (9.96)		
**Number of days part of program at time of census**	496.09 (1,555.37)		
**History of reported missing incidents**		21.51	182
**History of reported missing incidents before joining the program**		17.61	149
**History of reported missing incidents after joining the program**		7.21	61
**Diagnosis (*n* participants with history of missing incidents)**			
** Alzheimer’s disease**		36.76 (36.81)	311 (67)
** Vascular dementia**		23.05 (22.53)	195 (41)
** Mixed dementia**		20.69 (21.43)	175 (39)
** Unspecified dementia**		13.00 (16.48)	110 (30)
** Frontotemporal dementia**		3.31 (2.20)	28 (4)
** Dementia with Lewy bodies**		2.36 (0.55)	20 (1)
** Parkinson’s dementia**		0.83 (0)	7 (0)
**Season of missing incident**			
** Spring**		26.81	63
** Summer**		21.70	51
** Autumn**		28.94	68
** Winter**		22.55	53
**Time of day when missing reported**			
** Morning**		34.04	80
** Afternoon/Evening**		50.21	118
** Night**		15.74	37

*Note*. Percentages within diagnosis relate to the percentage of missing incidents in the overall sample.

**Figure 1.**
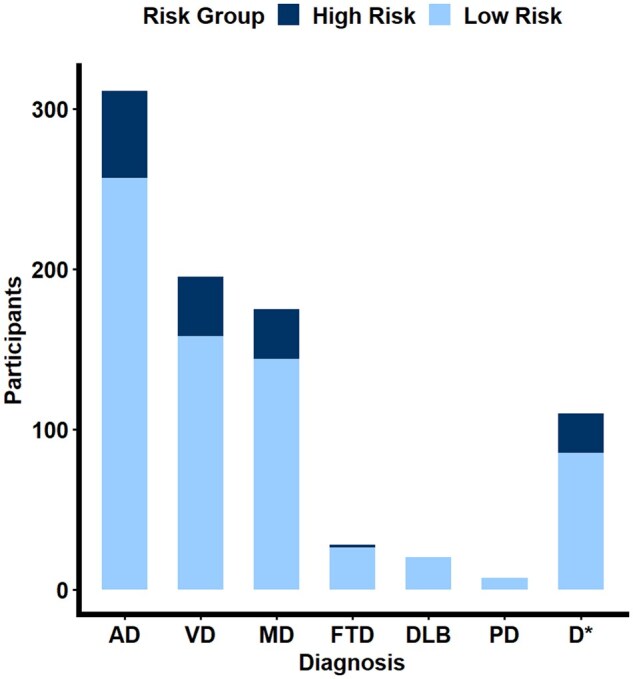
Grouped bar plot showing safeguarding risk status by dementia diagnosis. AD = Alzheimer’s disease; D = unspecified dementia; DLB = dementia with Lewy bodies; FTD = frontotemporal dementia; MD = mixed dementia; PD = Parkinson’s disease; VD = vascular dementia.

The majority of individuals who take part in the safeguarding scheme have a diagnosis of AD (36.76%), followed by VD (23.05%), mixed dementia (20.69%), an unspecified dementia diagnosis (13.00%), frontotemporal dementia (3.31%), dementia with Lewy bodies (2.36%), and Parkinson’s dementia (0.83%) (see Figure 1).

Of the high-risk group, the majority of individuals had AD (36.24%), followed by VD (24.83%), mixed dementia (20.81%), unspecified dementia (16.11%), and frontotemporal dementia (1.34%).

### Characteristics of missing incidents

The majority of missing incidents took place at home (86.08%), followed by in public (6.75%), a care home (5.49%), and a hospital (1.69%). The majority of individuals were found in public (85.59%), followed by at home (13.98%), and in a care home (0.42%).

Statistically significant differences were found in the time of day missing incidents were reported (χ^2^ = 41.93, df = 2, *p* <. 001). Bonferroni-adjusted post-hoc pairwise comparisons revealed that people were more likely to go missing in the afternoon/evening (50.21%) than in the morning (34.04%, *p* = .001) and night (15.74%, *p* = <.001). People were also more likely to go missing in the morning than at night (*p* <.001).

Most missing incidents took place during Autumn (28.94%), followed by Spring (26.81%), Winter (22.55%), and Summer (21.70%). There was no statistically significant difference in the season in which missing incidents were reported (χ^2^ = 3.35, df = 3, *p *= 0.34).

### Does the safeguarding scheme reduce missing incidences among people living with dementia?

**Figure 2.**
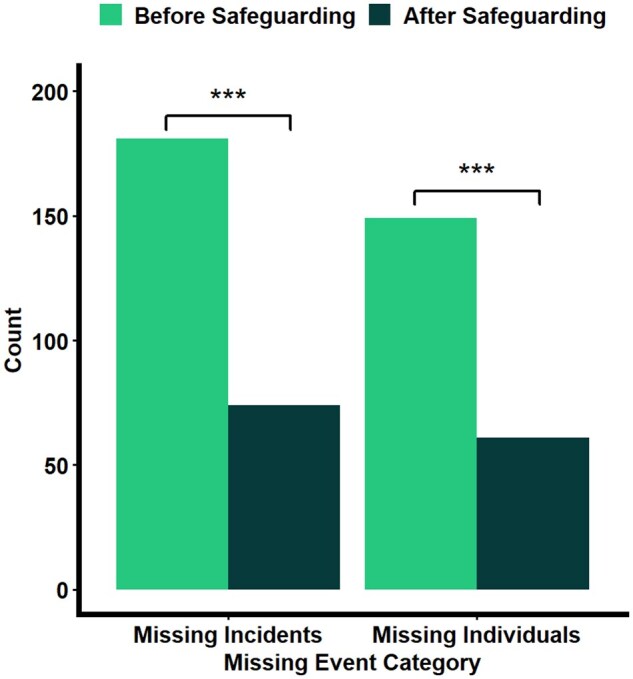
Frequency plots of missing incidents and the number of missing individuals before and after joining the safeguarding program.

Within the overall sample, fewer missing incidents took place after joining the safeguarding scheme (*M *= 0.09, *SD* = 0.34) than before the scheme (*M *= 0.21, *SD* = 0.52), *t*(1455.6) = 5.923, *p* < .001 (see Table 2). This was maintained when taking into account only individuals who joined the safeguarding scheme within 1 year prior to the data census, *t*(838.55) = 3.957, *p* < .001 (see Figure 2).

**Table 2.** Sample characteristics of missing incidents before and after joining the safeguarding program.

Variable	Pre-scheme	Post-scheme	Total	*p*	Effect size
**N. of missing individuals**	149	61	182	<.001	0.57
**Total number of missing incidents**	181	74	255	<.001	0.23
**Average duration of missing incident (hours)**	5.05 (11.30)	3.27 (3.20)	4.71 (10.39)	.10	0.13
**Average duration of first missing incident (hours)**	5.39 (12.57)	2.73 (1.92)	4.86 (11.32)	.04	0.24
**Location missing (home/public/care home/hospital)**	156/12/6/3	48/4/7/1	204/16/13/4	.11	0.16
**Location found (home/public/care home/hospital)**	28/147/1/0	5/55/0/0	33/202/1/0	.40	0.10

*Note*. Cohen’s *G* effect size used for analysis relating to number of missing individuals, missing incidents, and duration of missing incidents. Cramér’s *V* effect size used for location analysis.

### Does the safeguarding scheme reduce the risk of missing incidents in high-risk and low-risk individuals?

A total of 28 high-risk individuals recorded at least one missing incident after joining the safeguarding scheme (18.79%), compared to 33 low-risk individuals (4.73%). High-risk individuals were significantly more likely to record a missing incident after joining the safeguarding scheme than low-risk individuals (χ^2^ = 34.19, df = 1, *p* < .001). This was maintained when taking into account only individuals who joined the safeguarding scheme at least 1 year prior to the data census (χ^2^ = 48.92, df = 1, *p* < .001).

High-risk individuals were significantly less likely to record at least a missing incident after joining the safeguarding scheme than before joining (χ^2^ = 58.05, df = 1, *p* < .001). This was maintained for individuals who had joined the safeguarding scheme at least 1 year prior to the data census (χ^2^ = 16.49, df = 1, *p* < .001).

### Effect of the safeguarding scheme on the duration of missing incidents

There was no significant difference in the length of time missing for reported missing incidents before the safeguarding scheme (*M* = 5.05, *SD* =11.27) and after joining (*M* = 3.27, *SD* = 3.20); *t*(178.37) = 1.648, *p *= 0.10.

Within the high-risk group only, there was also no significant difference between the length of time missing before and after joining the safeguarding scheme, *t*(123.06) = 1.332, *p *= .19.

When looking at the difference in missing incident duration before and after joining the safeguarding scheme, only for first-time missing incidents, missing incidents were longer in duration before joining the scheme (*M *= 5.39, *SD* = 12.57) than after joining (*M *= 2.73, *SD* = 1.92); *t*(124.66) = 2.113, *p *= .04.

### Safeguarding scheme status and locations missing/found

There was no significant difference in location missing (*p *= .11) or location found (*p *= .40) between incidents occurring before and after joining the safeguarding scheme.

### Safeguarding scheme effectiveness and dementia diagnosis

There was no statistically significant difference between the number of missing incidences between individuals with AD (*M *= 0.20, *SD* = 0.47) or VD (*M *= 0.27, *SD* = 0.67) in the number of missing incidences reported before joining the safeguarding scheme. However, individuals with AD were more likely to have a missing incident (*M *= 0.11, *SD* = 0.40) than VD individuals (*M *= 0.04, *SD* = 0.20) after joining the safeguarding scheme (*t*(484.85) = 2.575, *p *= .01).

**Figure 3.**
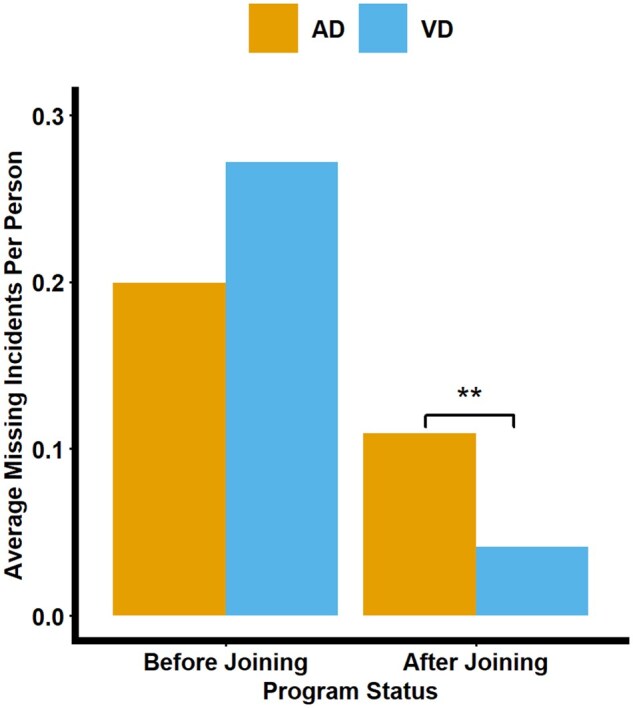
Bar plot showing the average number of missing incidents by dementia diagnosis before and after joining the safeguarding program. AD = Alzheimer’s disease; VD = vascular dementia.

There was no statistically significant difference in the number of individuals who went missing multiple times between the AD and VD groups (χ^2^ = 0.04, df = 1, *p *= .83) (see Figure 3).

There was no significant difference in the length of time missing between AD and VD individuals before (*t*(36.04) = −1.5281, *p *= .14) or after joining the safeguarding scheme (*t*(16.48) = 1.63, *p *= .10).

## Discussion

Overall, this study demonstrates that the safeguarding scheme implemented by the Avon and Somerset Police in the United Kingdom is effective in safeguarding against the risk of missing incidents for people living with dementia. Importantly, only 7.21% of individuals with dementia reported a missing incident after joining the safeguarding scheme, compared to 17.61% before joining the safeguarding scheme. While it was found that high-risk individuals (those who reported a missing incident prior to joining the safeguarding scheme) were more likely to go missing after joining the safeguarding scheme than low-risk individuals, 81.21% did not report a missing incident after taking part in the safeguarding scheme. From our findings, we therefore recommend that individuals living with AD, VD, and mixed dementia—who made up 80.50% of the present sample and account for between ∼80%–90% of dementia diagnoses—are offered to partake in the safeguarding scheme to reduce the probability of missing incidents.

Importantly, we found that 21.51% of individuals with dementia within the overall dataset reported a missing incident, and 5.79% of our study sample demonstrated repeated missing incidents. Significant variance has been found in the prevalence of missing incidents and repeat missing incidents in dementia across studies. Our findings of repeat missing incidents are in support of previous findings in the United Kingdom showing that the number of individuals who repeatedly go missing is approximately 5%–7% (McShane et al., 1998; White & Montgomery, 2015). Recent findings from Canada are higher, showing that repeat missing incidents were 22.50% (Miguel-Cruz et al., 2024). Differences found across studies may reflect unique characteristics of the study sample, and this international variance may also reflect geographical and environmental differences that affect missing incident risk. This requires exploration in the future.

Importantly, individuals who experienced a missing episode after joining the safeguarding scheme were found more quickly on average compared to before joining the safeguarding scheme. This difference did not reach statistical significance, which may reflect reduced analytical power due to smaller numbers of post-scheme missing incidents. When looking only at the difference in duration for first-time missing incidents that took place before and after joining the safeguarding scheme, a statistically significant difference was observed, with low-risk individuals being found significantly faster (2.73 average hours, ∼ 164 min) than high-risk individuals (5.39 hours ∼ 323 min). This discrepancy is potentially due to biases in considering the average duration for all missing incidents, as individuals with repeat missing incidents may experience different contextual factors that affect missing incident duration (e.g., greater carer vigilance). Restricting analysis to only the first missing incident duration provides a more accurate assessment of the safeguarding scheme’s efficacy in reducing the duration of missing incidents. Previous findings have shown that the median duration of dementia-related missing incidents in those who sustained harm was 3.55 hours (White & Montgomery, 2015), and duration missing is the strongest predictor of harm for people living with dementia (Larsson et al., 2025; Lissemore et al., 2019). When taking into account these findings, the Avon and Somerset Safeguarding Scheme is effective in reducing the risk of harm as an outcome of missing incidents.

Interestingly, while there were no significant differences between individuals with AD and VD in the prevalence of missing incidents prior to joining the safeguarding scheme, we found that individuals with AD were significantly more likely to go missing after joining the scheme compared to individuals with VD. This divergence may be attributable to the distinct cognitive profiles associated with AD and VD subtypes. AD is strongly associated with early and progressive impairments in spatial navigation and orientation, which are critical for independent wayfinding. These impairments can result in individuals with AD becoming disoriented even in familiar environments, which increases their risk of becoming lost, particularly during unsupervised outings (Coughlan et al., 2018). Spatial disorientation is much less established in VD, although see Lowry et al. (2023). Hence, it is not clear how vascular pathophysiology might contribute to spatial disorientation in VD. In addition, people with VD might have incipient AD pathophysiology, even without a mixed dementia diagnosis, which makes the delineation of spatial disorientation problems challenging. It highlights the need for further dementia- and person-specific risk-reduction strategies that address the specific cognitive vulnerabilities of people with dementia.

Another novel aspect of our study is the inclusion of information on the locations where individuals were reported missing and subsequently found, which is crucial to informing both prevention and response strategies. We found that most missing individuals (85.59%) were found in public locations, which aligns with the understanding that people with dementia may become disoriented during unsupervised outdoor outings. This finding is consistent with previous research, which similarly reported that 87% of missing incidents occurred in public places (Miguel-Cruz et al., 2024). Additionally, we found that 13.98% of individuals were found at home, suggesting that some may have returned independently or received assistance to safely return—a pattern also observed in previous studies (Larsson et al., 2025; Okita et al., 2016). These findings underscore the importance of safeguarding strategies tailored for community-dwelling people with dementia, enabling people to remain in their own homes. Maintaining independence while ensuring safety requires a nuanced understanding of where and how missing incident episodes occur. Future data collection should therefore aim to increase granularity in missing incidents information, such as examining the specific nature of public spaces—for example, differentiating between low-risk (e.g., supermarket) and high-risk (e.g., unfamiliar urban areas) places. It is important to establish more precise locations as previous findings demonstrate an association between increased risk of dementia-related missing incidences and environmental risk factors (Puthusseryppady et al., 2020).

It is important to note that the safeguarding scheme’s database captures only those incidents formally reported as missing and does not account for unreported episodes of spatial disorientation that do not escalate to police involvement. Consequently, it is possible that the number of missing incidents reported in this study for people living with dementia is an underestimation of the true incidence of missing events. Capturing data on the full spectrum of disorientation, including near-miss events, is essential for understanding how environmental features contribute to spatial disorientation and for shifting toward a proactive, prevention-based approach to reducing missing incidents (Puthusseryppady et al., 2022). Furthermore, both participation in the program and reporting of missing incidents may be influenced by stigma and equity perceptions (e.g., dementia-related stigma; access differences), which may influence involvement with the dementia safeguarding scheme. Future research should therefore also establish attitudes toward involvement in health policing interventions to ensure that the intervention is effectively engaging relevant communities.

Aligning with previous research, we found that the majority of missing incidents took place during the daytime (Larsson et al., 2025; White & Montgomery, 2015). While the majority of missing incidents took place in the autumn, no significant difference was reported in the number of missing incidents reported in each season. Previous studies have noted that missing incidents in colder seasons were found to be associated with increased risk of harm (Larsson et al., 2025; White & Montgomery, 2015). Although we were unable to directly measure outcomes related to harm, these seasonal patterns underscore the importance of proactive safeguarding during colder months to reduce the risk of harm in dementia-related missing incidents.

The findings that the safeguarding scheme reduces the risk of missing incidents among people with dementia has important repercussions for alleviating burden on family and carer stressors, as well as demand on emergency and health services. Avon and Somerset Police conservatively estimate the cost of police-led search operations for each dementia-related missing incident at approximately £2,500. Therefore, as well as reducing burden on family and carers, the safeguarding scheme has the potential to reduce financial and operational pressures on police services and the broader public sector. These findings demonstrate the program’s value not only as a protective intervention but also as a cost-saving public health measure.

Our study is not without limitations—largely due to dataset restrictions. The dataset does not include relevant demographic or diagnostic information enabling exploration into the risk factors of dementia-based missing incidents, such as sex, proximity to carers and living circumstances, environmental setting (urban vs rural), or disease staging. We were also not able to ascertain how long it took for individuals to be involved in the safeguarding scheme after receiving their dementia diagnosis. For future research exploring risk factors of missing episodes, it would be useful to include more granular location details, as well as information as to whether/how the individual had experienced any harm. Greater contextual information regarding the circumstances surrounding each missing incident would enable for a more comprehensive understanding of the cause of missing incidents, which can promote appropriate prevention strategies. Furthermore, it was not possible to determine from the database which participants were allocated a GPS device, and therefore whether device allocation significantly affected missing incident characteristics. It would be useful for future research to evaluate which of the devices are most effective in reducing missing incident occurrences and duration. Finally, to more robustly assess the impact of the safeguarding scheme on dementia-related missing incidents, the inclusion of a matched historical control group for comparison would enable more accurate determination as to whether the change in missing incident events is attributable to the safeguarding scheme or other contextual factors.

Beyond our specific findings, this study has broader dementia safeguarding practice and research implications. From a service delivery perspective, this study can inform potential strategies to efficiently inform allocation of police resources, including the economic cost of missing incidents, risk stratification for individuals with a greater likelihood of missing incidents, as well as identification of common times and locations of missing episodes. Targeted training for police personnel may further increase the impact of the safeguarding scheme. From a research perspective, this study highlights the importance of analyzing first-time missing incidents individually to reduce bias in evaluation. Future research should also address factors such as accurate estimation of disorientation events in people living with dementia and more specific, comprehensive databases for missing dementia safeguarding databases. Given the success of the current safeguarding scheme, research should also address potential barriers to access to ensure the safeguarding scheme is efficiently reaching relevant population groups before missing incidents occur. In addition, longitudinal follow-up studies and comparative evaluations with other police-health collaborations can assist in identifying the most effective practices and developing unified approaches across regions.

In conclusion, our findings demonstrate that the Avon and Somerset Dementia Safeguarding Scheme is effective in improving the safeguarding of people with dementia for missing incidents. In turn, this will allow people with dementia to remain safer and independent for longer. It will also provide reassurance to carers and families of people with dementia that an effective safeguarding scheme can reduce the likelihood of missing incidents, as well as the demand and cost burden on public services. Finally, our findings support the widespread use of proactive, Police-led safeguarding programs to improve safety and independence for people living with dementia.

## Supplementary Material

igaf132_Supplementary_Data

## Data Availability

The dataset used for analysis will not be made publicly available due to the sensitive nature of the Dementia Safeguarding Scheme database. The study was not preregistered.
