# Supplementary material for: Impact of a police safeguarding program on reducing dementia-related missing incidents in the United Kingdom
Source: Innov Aging. 2026 Mar 4;10(3):igaf132. doi: 10.1093/geroni/igaf132 (PMC12962802; doi:10.1093/geroni/igaf132)
Supplement: igaf132_Supplementary_Data [file igaf132_supplementary_data.docx]

# **Supplementary Material**

**Supplementary Table 1: Description of RFID devices provided by Avon and Somerset Police Safeguarding Scheme**

| **Device:** | **Description:** |
| --- | --- |
| GPS tracking pendant | Individuals most at-risk of missing incidents (those who had previously recorded a missing incident) were allocated GPS tracking pendants. GPS pendants enable real-time location tracking for individuals identified as being at risk of going missing, enabling caregivers to monitor their movements and quickly locate them if they become lost. |
| NFC Assistive Device | Wristbands and hangtags storing essential identification and contact information can be accessed by scanning an embedded chip, allowing members of the public or emergency services to assist a person with dementia by quickly identifying them and taking appropriate action. |
| Tile trackers | Tile trackers utilise Bluetooth and Life360’s global network to help locate missing items or individuals by connecting to nearby smartphones, displaying contact details when scanned, and working within a range of up to 500 feet. Tile devices also have a QR code which displays details about the wearer and their next of kin. |
